# Fabrication of 3D Printed Poly(lactic acid)/Polycaprolactone Scaffolds Using TGF-β1 for Promoting Bone Regeneration

**DOI:** 10.3390/polym13213731

**Published:** 2021-10-28

**Authors:** Cheng-Hsin Cheng, Ming-You Shie, Yi-Hui Lai, Ning-Ping Foo, Mon-Juan Lee, Chun-Hsu Yao

**Affiliations:** 1 Graduate Institute of Medical Sciences, Chang Jung Christian University, Tainan 71101, Taiwan; u701018.tw@yahoo.com.tw (C.-H.C.); ningping.tw@gmail.com (N.-P.F.); 2 Department of Neurosurgery, An Nan Hospital, China Medical University, Tainan 70965, Taiwan; 3 3D Printing Medical Research Center, China Medical University Hospital, Taichung 40402, Taiwan; gold018@gamil.com.tw; 4 School of Dentistry, China Medical University, Taichung 40402, Taiwan; 5 Department of Bioinformatics and Medical Engineering, Asia University, Taichung 40402, Taiwan; 6 Department of Biomedical Imaging and Radiological Science, China Medical University, Taichung 40402, Taiwan; gold8701018@gmail.com; 7 Department of Emergency Medicine, An Nan Hospital, China Medical University, Tainan 70965, Taiwan; 8 Department of Bioscience Technology, Chang Jung Christian University, Guiren Dist., Tainan 711301, Taiwan; 9 Department of Medical Sciences Industries, Chang Jung Christian University, Guiren Dist., Tainan 711301, Taiwan; 10 School of Chinese Medicine, China Medical University, Taichung 40402, Taiwan; 11 Biomaterials Translational Research Center, China Medical University Hospital, Taichung 40402, Taiwan; 12 Department of Biomedical Informatics, Asia University, Taichung 40402, Taiwan

**Keywords:** bone regeneration, polycaprolactone, poly-lactic acid, three-dimensional printing, transforming growth factor-β

## Abstract

Our research was designed to evaluate the effect on bone regeneration with 3-dimensional (3D) printed polylactic acid (PLA) and 3D printed polycaprolactone (PCL) scaffolds, determine the more effective option for enhancing bone regeneration, and offer tentative evidence for further research and clinical application. Employing the 3D printing technique, the PLA and PCL scaffolds showed similar morphologies, as confirmed via scanning electron microscopy (SEM). Mechanical strength was significantly higher in the PLA group (63.4 MPa) than in the PCL group (29.1 MPa) (*p* < 0.01). Average porosity, swelling ratio, and degeneration rate in the PCL scaffold were higher than those in the PLA scaffold. SEM observation after cell coculture showed improved cell attachment and activity in the PCL scaffolds. A functional study revealed the best outcome in the 3D printed PCL-TGF-β_1_ scaffold compared with the 3D printed PCL and the 3D printed PCL-Polydopamine (PDA) scaffold (*p* < 0.001). As confirmed via SEM, the 3D printed PCL- transforming growth factor beta *1* (TGF-β_1_) scaffold also exhibited improved cell adhesion after 6 h of cell coculture. The 3D printed PCL scaffold showed better physical properties and biocompatibility than the 3D printed PLA scaffold. Based on the data of TGF-β_1_, this study confirms that the 3D printed PCL scaffold may offer stronger osteogenesis.

## 1. Introduction

Currently, the demand for bone graft materials is increasing significantly [1]. Research on several forms of bone substitutes has been ongoing for years concerning the repair of defects [2,3,4]. Important factors that are considered for an ideal bone substitute material are the capability to transport cells, induce differentiation of osteoblasts, biocompatibility, osteoinductivity, osteoconductivity, proper biodegradation, and the development of osteoblasts in the defective space [5,6,7].

Additive manufacturing (AM) may be an option when traditional manufacturing has limitation of application. Rapid prototyping, functional integration, lightweight design, a design policy for high degree freedom have been developed. The technique of AM can be applied not only for production using other optimized methods in design but also prototyping including ideal support, ideal topology and options of partial consolidation and partial orientation [8]. AM procedures are also known as 3-dimensional (3D) printing, an improvement of techniques that decades ago permitted the construction of flat pieces from digital files [9]. The 3D printed structures are manufactured layer-by-layer with an injected cement or biomaterials [10,11]. The benefits of 3D printed frameworks are designable porosity, structure pore shape, and morphology, as compared with conventional procedures [12]. 3D biomedical devices are critical in tissue engineering because of the ability to design and fabricate complexes. Custom-made transplants can be made with precisely designed constructions according to the 3D image data of patients, which offers great superiority over traditional procedures for manufacturing 3D porous scaffolds, such as salt-leaching/ solvent-casting, air jet spinning, gas foaming, and electrospinning techniques [13,14,15,16]. Current 3D printing procedures offer opportunities for better conventional bone substitutes, providing sufficient pore interconnection, pore shape, and optimal porosity [17].

Polylactic acid (PLA) is one of the substances in our 3D printed scaffold. PLA is a bio-based material presenting fascinating physical, chemical and mechanical properties such as sufficient biodegradability, simple processing, and high ductility and hardness [18]. However, it displays some limitations such as poor water solubility, and being frangible [19]. Owing to its good accessibility, PLA is currently one of the most broadly applicable biodegradable polymers for various uses in packaging, electronic industries, automotive industries, and prosthesis fabrication, substituting effectively for petroleum-based polymers [20,21]. Many trends in orthopedic practice and research have suggested that PLA is safe and nontoxic [22]. Numerous studies have demonstrated the biocompatibility of these biomaterials in vivo and in vitro [23]. In a study, appropriate tissue biocompatibility was reported when PLA was used as a graft in the femoral shaft of sheep [24]. PLA and its substitutes have also been commonly applied in bone repair and have shown to have sufficient biological adaptation, be nontoxic, and noninflammatory [25]. Transplants made of PLA were shown to enhance bone repair in rats and create better bone regeneration than controls in skull defects [26].

Polycaprolactone (PCL) is another material in our 3D printed scaffold. As a biomaterial for scaffold construction, PCL offers several advantages, such as adjustable biodegradation and relatively high mechanical properties at physiological temperatures [27]. The biocompatibility and degradation of PCL can be modified by merging it with other biomaterials to develop composite biomaterials for bone repair [28]. With the combination of copolymerization, or mixing with other substances, PCL properties can be improved. PCL has sufficient hardness and mechanical stiffness under physiological conditions owing to its poor glass transition temperature and semicrystalline ability [25,29]. For efficient tissue engineering, the surface of the PLA framework is often modified with hydrophilic substances to enhance cell binding and growth. However, owing to its slow degeneration, PCL is combined with other polymers and ceramic substances to modify its degradation for a specific application [30]. 

MG-63 cell is one of the osteosarcoma cell lines. All investigated osteosarcoma cell lines (e.g., MG-63, Saos-2 and U-2 OS) exhibit very heterogeneous labelling profiles and each differs from typical osteoblasts. The osteosarcoma cell line displays a characteristic labelling profile that offers a differently composed extracellular matrix. This can be used to better characterize osteosarcoma, as well as for its diagnosis [31]. In one MG-63 cell study, proliferation was more rapid than in other osteoblast-like cells with a short doubling time. MG-63 cells also had increased enzyme activity following 1,25-dihydroxyvitamin D3 (1,25(OH)2D3) administration, which is characteristic of bone-derived cells [32]. This cell line was used for the study of bone cell culture and was effective in bone growth.

In the production of scaffolds with appropriate physical properties, 3D printing is the most adopted methodology [33]. Although 3D printed scaffolds have exhibited acceptable bone tissue repair, the performance of synthetic substances is usually affected by many biological factors [34]. The advantage of 3D printing lies in facilitating the design of a variety of different configurations of the skeleton. To enhance bone regeneration with 3D printed scaffolds, we used dopamine (DA)-mediated immobilization for transforming growth factor beta 1 (TGF-β_1_)-derived peptides onto biodegradable 3D printed scaffolds to promote bone regeneration. DA has a catechol functional structure that can increase polymerisation through oxidative transformation from catechol to quinone [35,36]. Polydopamine (PDA) can cover the surface of the scaffold as a thin layer [36,37]. Many researchers have shown that thiolated polysaccharides, peptides, enzymes, growth factor proteins, and anticancer drugs (e.g., neurotrophic and angiogenic abilities) can be efficiently grafted onto DA-coated material structures [38,39]. The role of TGF-β_1_ is crucial for bone formation during the stages of cell proliferation, differentiation, and promotion of osteogenesis [40,41]. In this study, osteoinductive peptides derived from TGF-β_1_ could be effectively fixed onto 3D printed scaffolds through the PD-mediated coating technique [42]. 

The objectives of our study are to explore the biological and physical properties of 3D printed PLA and PCL. As we know, 3D printing is advantageous because it provides an appropriate structure. However, different materials may have different effects on bone repair. Our aim is to improve bone regeneration and develop 3D printed scaffolds with better artificial biomaterials for artificial bone.

## 2. Material and Methods

### 2.1. Creation of 3D Printed PLA and PCL Scaffolds

Each 3D printed scaffold was devised with the SpaceClaim 2014 CAD package (SpaceClaim Corporation, Concord, MA, USA). In the 3D printing machine, a cartridge was applied to supply the feedstock filament of polymers (Pitotech, Changhua City, Taiwan) into the fused deposition modeling (FDM) 3D printer (Prusa I3). The PLA or PCL strand was drawn, merged and extruded over the printing head to layer beads. The printer had the ability to mix up to three separate threads with a diameter of 0.2 mm. The thickness of each layer for the designed scaffolds was set to 0.2 mm in order to achieve better support and permeability with good printing quality. The scaffold was set with a cylindrical outline with a height of 8 mm and a diameter of 6 mm.

### 2.2. Analysis of Physical Property 

#### 2.2.1. Mechanical Testing

Mechanical compression was measured with a SHIMADZU (AG-10KNIS) testing device using a 10 kN load cell according to the guiding procedure set in ASTMD5024-95a [43]. Each group of data was tested for five samples (*n* = 5).

#### 2.2.2. Measurement of Porosity

The specific gravity method was used to test porosity. At a temperature of 37 °C, the scaffold (m_s_) was immersed in ethanol (m_0_), making sure all pores in each sample were filled with ethanol (m_1_), then the soaked specimen was removed. The remaining weight of the ethanol and specific gravity bottle was recorded as m_2_. Porosity was calculated as Porosity = (m_1_ − m_2_ − m_s_)/(m_0_ − m_2_).

#### 2.2.3. Measurement of Swelling Ratio 

The samples were fully immersed in Sorensen’s buffer fluid. After immersion for different time points at 37 °C, the samples were taken out, air-dried with filter paper, and weighed (*W*_t_). Next, these scaffolds were frozen, dehydrated, and weighed (record as W_0_). The swelling percentage (Δ*W*%) was calculated as Δ*W*(%)  =  (*W*_t_  −  *W*_0_)/*W*_0_ × 100(%) [44].

#### 2.2.4. Degradation Rate Measurements

The rate of degradation was estimated under ISO 10993 guidelines. All the studied scaffolds were weighed (record as W_0_) and put into testing tubes with 3 mL of Sorensen buffer. Each sample tube was left at 37 °C. At scheduled time points after soaking, all sample scaffolds were removed from the Sorensen buffer, freeze-dried, and weighed (recorded as *W*_t_). The weight loss percentage (recorded as ∆*W%*) was then calculated (Δ*W*% = (*W*_0_ − *W*_t_)/*W*_0_ × 100%). Each rate of degradation was defined by dividing its ∆*W%* by the different soaking time point.

### 2.3. Analysis of Biocompatibility In Vitro

#### Stem Cells Culture by Scanning Electron Microscopy (SEM) Observation

MG-63 cells (BCRC, number 60279) were cocultured with scaffolds as a single layer in low-glucose Dulbecco modified Eagle medium (DMEM) (D6046, Sigma, Taipei, Taiwan) with 1% PS and 10% FBS under 37 °C, 5% CO_2_, and 95% air in a humidified setting. After the setup time point, seeded scaffolds were irrigated several times by 1× phosphate buffered saline (PBS), immobilized in 2% glutaraldehyde for 48 h, and critical point dehydrated. Then, the samples were sputter-covered with platinum for 200 s by vacuum and observed by SEM (S-3000; Hitachi, Tokyo, Japan) at 15 kV.

### 2.4. Coating Poly(dopamine) and Immobilized TGF-β_1_

Coating of PDA (H8502, Sigma, Taipei, Taiwan) on the surface of PCL scaffold sample was performed using an immersion coating procedure. The PCL scaffold was placed in 0.5 mL DA fluid (pH 8.5, 2 mg/mL in 10 mM Tris, H8502, Sigma, Taipei, Taiwan) with a shaker at 25-rpm at 37 °C for 12 h, followed by several washes with deionized water according to our previous study [45]. The PDA-coated PCL scaffold samples were placed in the TGF-β_1_ fluid (pH 8.5, 10 or 20 ng/mL, 10 mM Tris-HCl buffer PeproTech, London, UK) and left at a temperature of 37 °C overnight.

#### 2.4.1. Quantification of Immobilized TGF-β_1_

We used an enzyme-linked immunosorbent assay (ELISA assay) (Cat.88-8350-88, Invitrogen) for measurement of TGF-β_1_ concentration on the scaffolds. For the preparation of different concentrations of TGF-β_1_ solution for the calibration curve, we conducted the TGF-β_1_ capture antibody reaction at 4 °C overnight. Aspirated wells were then washed three times by wash buffer solution and block by 200 μL ELISA Diluent (1X) at room temperature for 1 h. Aspirated wells were washed several times with wash buffer solution and 100 μL/well residual solution added for a reaction period of 2 h. Aspirated wells were irrigated three times by wash buffer, and rhBMP-2 added for 1 h for antibody. Aspirated wells were then washed three times with wash buffer, and avidin-HRP added for 30 min. The aspirated wells were then washed three to four times with wash buffer and reacted with 100 μL TMB for 15 min. The procedure was finished with 2 N HCl. An ELISA reader was used to read absorbance (450 nm). The interpolation method in the ELISA calibration curve was made to calculate the residual content of the TGF-β_1_ solution. This was calculated as immobilized concentration of TGF-β_1_ equals TGF-β_1_ concentration before reaction minus TGF-β_1_ concentration after reaction.

#### 2.4.2. TGF-β_1_ Release Test

We used an enzyme-linked immunosorbent assay (ELISA) to check the long-term release kinetics of TGF-β_1_ indirectly. For this step, supernatants were entirely assembled and cleaned by PBS solution on days 1, 3, 5, 7, 14, 21, and 28. The supernatants (1 mL) at different time point were preserved at −70 °C until the ELISA study.

#### 2.4.3. ALP Assay

MG-63 cells were placed onto 24 well cell culture plates at 1 × 10^4^ cells/well and 300 μL of sample scaffold soaking solution was added after 7 days. Thereafter, the medium was extracted and the stem cells were irrigated by PBS solution. A volume of 200 μL/well of ALP reagent (SIGMAFAST^TM^ pNPP substrate, Simga N2770) was placed in the dark for 30 min, and 405 nm wavelength absorption read using an ELISA detector.

#### 2.4.4. Cytotoxicity Testing

The (3-(4,5-dimethylthiazol-2-yl)-2,5-diphenyl tetrazolium bromide; Cat.298-93-1, USB) (MTT) assay was performed on the reduction of MTT using the mitochondrial enzyme (succinate dehydrogenase) to develop insoluble dark-blue formazan. 

Co-cultured cells constituted an osteoblast-like cell strand (MG-63 cell) from the Food Industry Research and Development Institute (FIRDI, Hsinchu, Taiwan). Osteoblasts cocultured with 3D printed scaffolds were used to check biocompatibility in vitro. The MG-63 cells were placed into 24 well cell culture dishes at 1 × 10^4^ cells/well, and 300 μL of 3D printed scaffold soaking solution incubator was added for 7 days. In this study, the scaffolds were immersed in 180 μL/well of culture solution and 20 μL/well of MTT medium, at a temperature of 37 °C for 4 h to develop crystals of formazan. The fluid was then extracted and 200 μL/well of acid propan-2-ol was dripped into all the wells and gently stirred to melt the dark-blue crystals. After a few minutes at 37 °C, the plates were detected by an ELISA machine (570 nm wavelength compared with a reference 650 nm wavelength). The accumulation of vital cells was recorded by converting the optical density (O.D) values according to the MTT study.

## 3. Results

### 3.1. Demonstration of PLA and PCL Scaffolds

**Figure 1:**
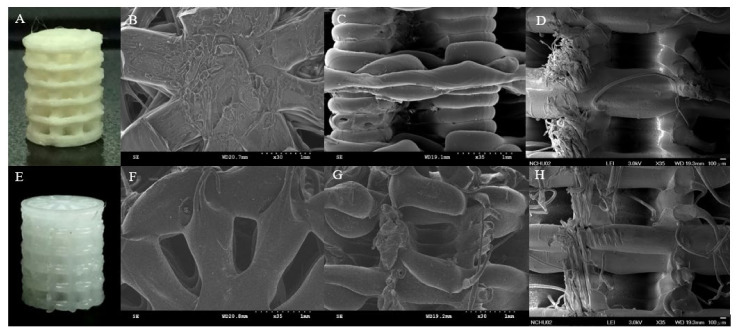
Morphology of 3D printed PLA and 3D printed PCL scaffold. The appearance of PLA (**A**) and PCL (**E**) scaffold. The top view, side view, and cross section of PLA (**B**–**D**) and PCL (**F**–**H**) scaffold under SEM study.

We developed 3D printed PLA and PCL scaffolds with similar shapes (Figure 1). Measured by SEM, the line width and pore size were almost the same in the 3D printed PLA and PCL scaffolds (Table 1). This showed that, even with different materials, the morphology was the same employing the 3D printed technique. For a better cell environment, an optimal scaffold microarchitecture should have maximum porosity with interconnected channels of ideal widths (approximately 200 to 900 μm in diameter) [46] and have a maximal surface area to permit sufficient rates of substance transfer, neo-vascularization and cell in-growth, [47]. The average pore sizes of our designed 3D printed scaffold were 684 μm or 682 μm. This is suitable for cell growth.

**Figure 2:**
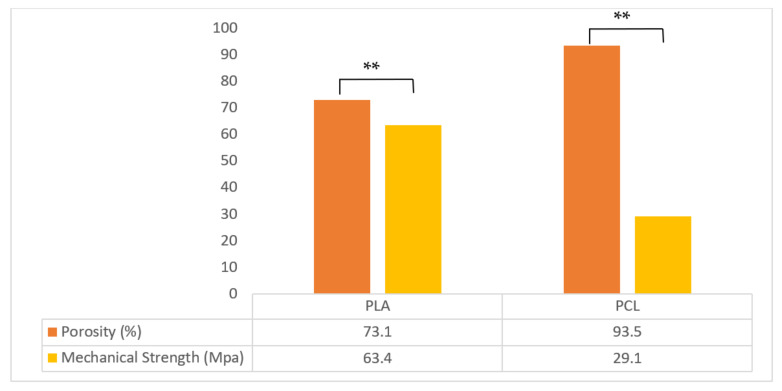
Porosity and mechanical strength of the 3D printed PLA and PCL scaffolds. Significantly different porosity and mechanical strengths are shown (** *p* < 0.01, *n* = 5).

The mechanical strength was significantly better in the 3D printed PLA scaffold than 3D printed PCL scaffold (Figure 2). With the same architecture of these two experimental scaffolds, the mechanical strength was influenced by the different material characteristics. The Young’s modulus of human cortical bone is from 1 to 20 GPa with a strength from 1 to 100 MPa [48]. The corresponding data for cancellous bone is 0.1 to 1.0 GPa and 1 to 10 MPa, respectively [49]. Although a wide range is reported for the mechanical strength of human natural bone, the values are references for determining the required mechanical properties of an ideal scaffold [50]. Our data show that both PLA and PCL scaffolds offer sufficient supportive strength.

**Figure 3:**
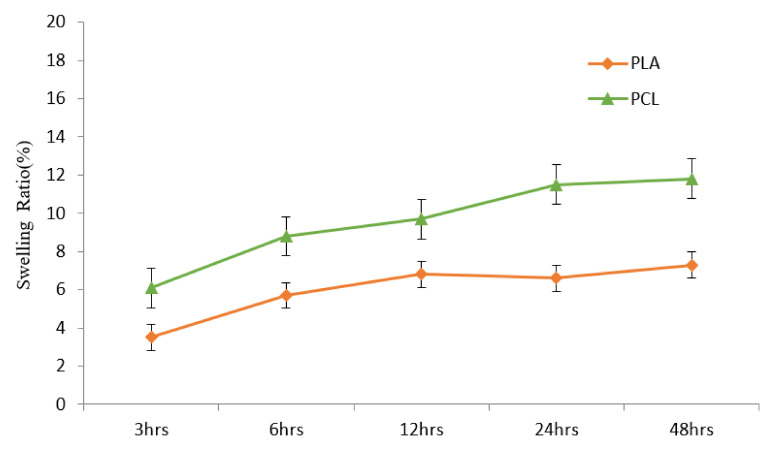
Swelling ratio of the 3D printed PLA and PCL scaffolds for 48 h of immersion. The swelling rate is higher in the PCL group after 48 h (*n* = 5).

**Figure 4:**
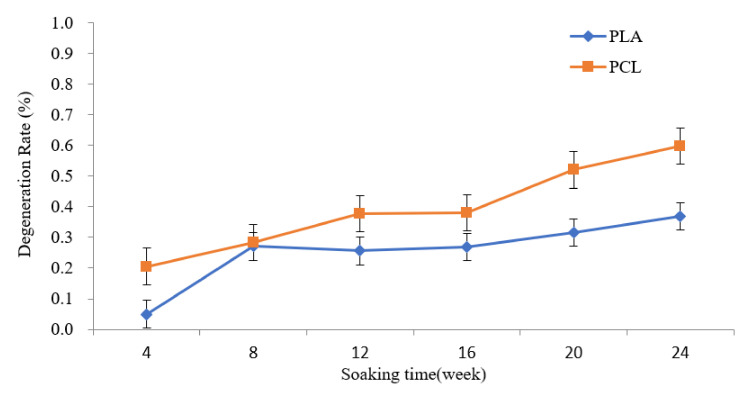
Degeneration rate of 3D printed PLA and PCL scaffolds for 24 h of immersion. The degeneration rate is higher in the PLA group (*n* = 5).

Porosity is related to the number of cell implants, connectivity of the pores, and strength of the structure [28]. The larger the porosity, more cells can be implanted. A larger porosity can offer better adherence and growth space. However, a larger porosity may cause weaker mechanical strength [51]. The porosity was 93.5% in the PCL scaffold and 73.1% in the PLA scaffold (*p* < 0.01) (Figure 3). In the study of swelling ratio, stability was attained after 24 h of immersion (Figure 4). The swelling ratio of PLA was lower than that of the PCL scaffold. According to the porosity, surface contact was greater in the PCL. Better surface contact may result in more water absorption and an advanced swelling ratio. Similar data were observed in the degradation test (Figure 4). The degradation rate was higher in the PCL scaffold than in the PLA group. These results may be the consequence of poor PLA biodegradability [52].

### 3.2. Biocompatibility

**Figure 5:**
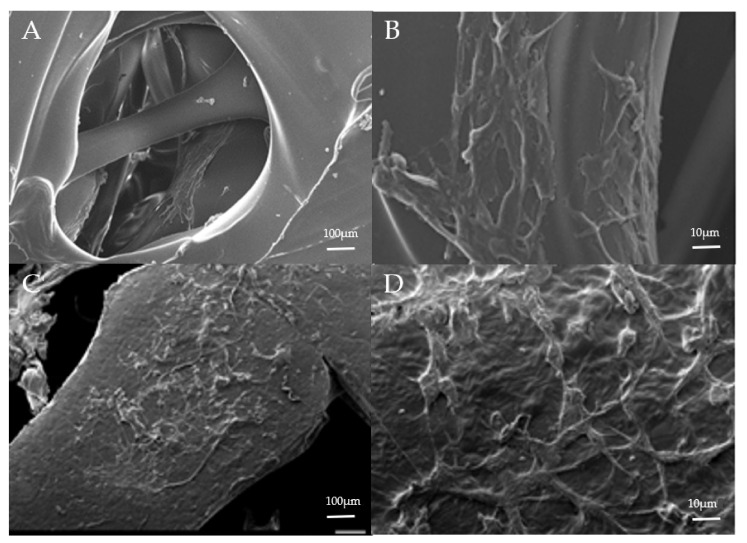
Cell culture in the 3D printed PLA (**A**,**B**) and PCL (**C**,**D**) scaffolds. Good cell adaption and adhesion were seen in both scaffolds. In gross view, more cell growth is seen in the PCL scaffold.

A cell culture study was prepared to estimate biocompatibility. MG-63 cells were cocultured with scaffolds for 24 h. The cells were observed by SEM (Figure 5). MG-63 cells were attached well to both 3D printed PLA and PCL scaffolds, producing pseudopodia, and showed increased cell adhesion and growth in the PCL scaffold. This implies that the 3D printed PCL scaffold is more favorable for cell growth than the PLA scaffold in vitro. However, the PLA may release lactate during degradation. A low pH may cause cell damage and poor cell attachment [53]. The 3D printed PCL in this study exhibited excellent biocompatibility.

### 3.3. Coating PDA and Immobilized TGF-β_1_

#### 3.3.1. Quantification of Immobilized TGF-β_1_

**Figure 6:**
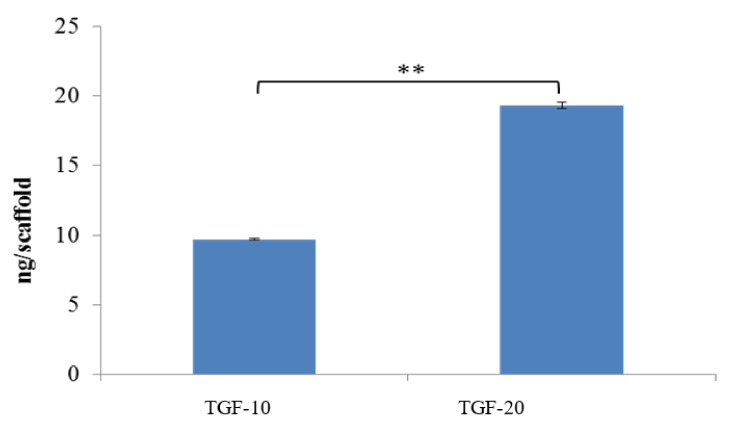
Concentration of TGF-β_1_ on the PCL-coated scaffold in two groups, proving the TGF-20 can offer more TGF-β_1_ concentration, (** *p* < 0.01, *n* = 5).

To identify the best concentration of TGF-β_1_ for PDA grafting, the PD-coated scaffold was immersed in two concentrations of TGF-β_1_ (Figure 6). The 10 ng/mL concentration of TGF-β_1_ is represented as TGF-10. The 20 ng/mL concentration of TGF-β_1_ is represented as TGF-20. The average levels of grafted TGF-β_1_ on the PDA-coated scaffold in TGF-10 and TGF-20 were 9.69 and 19.30 ng/scaffold. This implies that TGF-β_1_ could be fixed on the surface of the material through PDA connection. The concentration of immersed TGF-β_1_ was proportional to the level of fixed TGF-β_1_ on the scaffold. The fixation rates of the two groups reached above 50%. As mentioned above, we believe that, through PD grafting, TGF-β_1_ can be successfully fixed on the scaffold and has an appropriate fixed efficiency. 

#### 3.3.2. TGF-β_1_ Releasing Test

**Figure 7:**
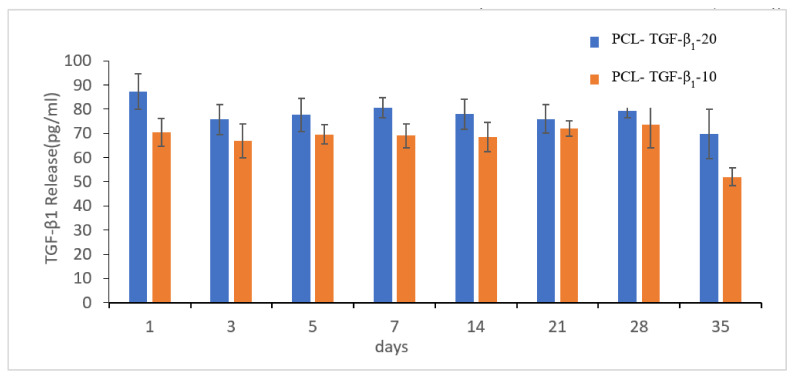
Analysis of TGF-β_1_ releasing level in the PCL-coated TGF-β_1_-10 (TGF-β_1_ concentration: 10 ng/mL) and PCL-coated TGF-β_1_-20 (TGF-β_1_ concentration: 20 ng/mL) samples at different time points (1, 3, 5, 7, 14, 21, 28, 35 days). The scaffold was able to release the TGF-β_1_ persistently and regularly for one more month. Moreover, the PCL-coated TGF-β_1_-20 produces a greater TGF-β_1_ level (*n* = 5).

**Figure 8:**
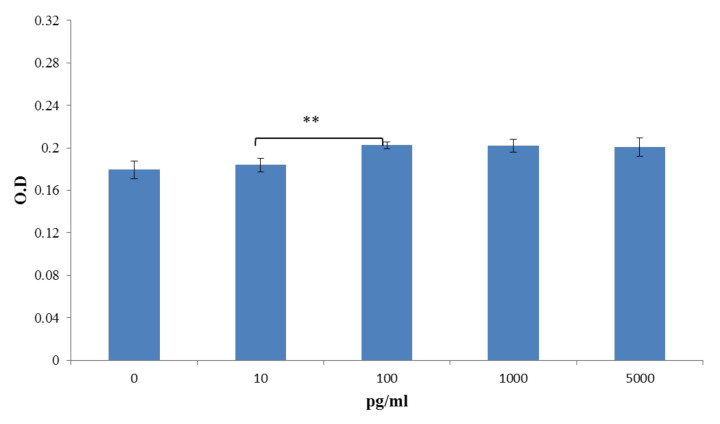
Optical density (O.D) of ALP assay by coculture with TGF-β_1_ and MG-63 cells for 5 days. A concentration of 100 pg/mL TGF-β_1_ has more influence on the ALP elevation, (** *p* < 0.01, *n* = 5).

We designed the study to analyze TGF-β_1_ releasing level with different concentrations of TGF-β_1_ grafting (10 ng/mL, 20 ng/mL) for 35 days in vitro (Figure 7). There was no abrupt release in the two groups within 7 days. The final accumulated TGF-β_1_ releasing levels in TGF-10 and TGF-20 for 35 days were 541.87 pg/mL and 621.355 pg/mL. The TGF-20 scaffold released more TGF-β_1_ (*p* > 0.001), implying that TGF-β_1_ could be slowly released from the scaffold over time. To confirm the effective concentration of TGF-β_1_ for bone growth, the ALP assay was evaluated by coculture with MG-63 cells with different levels of TGF-β_1_ (Figure 8). A quantity of 100 pg/mL TGF-β_1_ was most effective for ALP activity. Therefore, we compared TGF-β_1_ releasing levels at different time periods in the two groups. The effective concentration of TGF-β_1_ (100 pg/mL) was almost achieved in the TNF-20 group within 28 days. Considering cost and effectiveness, the best immersion concentration of TGF-β_1_ for the PDA-coated scaffold may be 20 ng/mL.

#### 3.3.3. MG-63 Culture by SEM Observation

**Figure 9:**
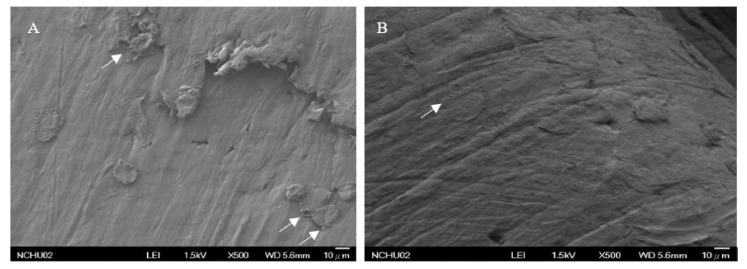
SEM observation of co-culture with MG-63 cells and the PCL-TGF-β_1_ scaffold for 6 h (**A**,**B**) showing good cell adhesion and typical cellular pseudopodia (white arrows).

MG-63 cells were implanted onto the 3D printed PCL-TGF-β_1_ scaffold for 6 h. Cell adhesion and growth were observed by SEM. The stem cells were polygonal and round-shaped on the scaffold after 6 h of culture (Figure 9). Therefore, the 3D PCL scaffold with PDA-coated and TGF-β_1_ grafting may be capable of enhancing cell transformation and growth.

#### 3.3.4. MTT Assay with MG-63 Culture

**Figure 10:**
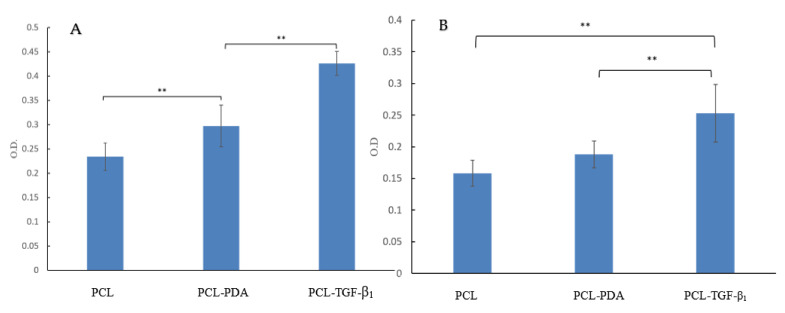
MTT assay after stem cell culture for 7 days (**A**). ALP assay after stem cell culture for 7 days (**B**). The PCL-TGF-β_1_ group had significantly lower toxicity and higher ALP level (** *p* < 0.01, *n* = 6).

An MTT assay was used to estimate degraded products on osteoblasts (Figure 10A). The cell count in the PCL-PDA and PCL-TGF-β_1_ groups was higher than the PCL group. This revealed that the degraded products of PDA and TGF-β may cause no toxicity and enhance cell activity. In addition, cell proliferation in the PCL and PCL-PDA scaffolds was better than that in the PCL-coated TGF-β_1_ group (*p* < 0.01). We believe that the 3D printed PCL-TGF-β_1_ scaffold has better biocompatibility because of PDA and TGF-β_1_ grafting during manufacturing [54].

#### 3.3.5. ALP Assay with MG-63 Culture

ALP is a protein commonly indicated for osteoblast differentiation [55]. We evaluated the ALP level on the cultured scaffold on the 7th day (Figure 10B). The ALP level was higher in the PDA-coated 3D printed PCL and PDA-coated TGF-β_1_ 3D printed PCL scaffold than in the pristine 3D printed PCL scaffold. This may show the ability of cell transformation and growth after PDA coating and TGF-β_1_ grafting. The highest ALP level was observed in the PCL-coated TGF-β_1_ group (*p* < 0.01). This may prove that the PDA-coated TGF-β_1_ 3D printed PCL scaffold has a better ability to enhance stem cell transfer to osteoblasts.

## 4. Discussion

The purpose of this research was to enhance the biological functionality of 3D printed synthetic scaffolds through coating with PDA and TGF-β_1_. Many studies have demonstrated the significance of PDA and TGF-β_1_ in bone regeneration [35,42,56,57]. However, the role and mechanism of 3D printed bone repair are not clearly understood [58]. We also attempted to evaluate the mechanism of bone repair in 3D printed scaffolds. Their capacity to induce the differentiation of newly seeded osteoblasts was also evaluated.

The 3D printing technique has lately been developed as a viable method for the construction of materials [59]. In particular, the melt extrusion printing method has allowed the deposition of polyesters frequently applied in biological engineering, such as PLA and PCL [60]. These polymers, and other generally extruded synthetic materials, are highly bioinert, demanding more deposition of bioactive substances [61]. The 3D printing method combined with biodegradable materials has been applied to create complicated structure for the repair of various organs or tissues, such as neural tissue, heart, cartilage and bone [62,63,64]. Therefore, 3D printing technology is superior to traditional methods. 

Two different materials with the same 3D printed structure (PLA and PCL) were developed to evaluate their physical properties. In the 3D printed PCL scaffold, based on physical evaluation, we found that the pore size was approximately 682 μm, the porosity was up to 93.5%, and the mechanical strength was less than that of the 3D printed PLA scaffold. Several studies have shown that high porosity with proper diameters from 300 to 900 μm in the scaffolds are ideal for tissue repair. These parameters of microarchitecture were able to offer a sufficient surface area to volume ratio to enhance neo-vascularization, cell growth and mass transfer [65]. However, porosity and interconnection in the frameworks may be associated with the mechanical strength of the architecture [66]. Consequently, porosity decreases with increase in the number of printed lines in each sheet. Previous reports stated that low mechanical strength of the scaffold may be caused by high porosity [45,67]. In our study, both PLA and PCL scaffolds displayed sufficient mechanical strength for bone support. Moreover, the data show the swelling ratio of PCL compared with the PLA scaffold. In relation to porosity, the PCL scaffold presented higher surface contact area. This greater superficial contact area may be result of stronger water absorption, inducing a better swelling ratio [39]. Thomas and Windle’s model of Case II diffusion verified water uptake is dependent on the pressure caused by swelling of the polymer. Hence, water uptake is strongly influenced by mechanical deformation of the glassy region in response to the swelling of polymer. In the bioassay in vitro, SEM showed that MG-63 cells had more pseudopodia and good cell attachment in the 3D printed PCL scaffolds. This may indicate the 3D printed PCL scaffold has better bone-regeneration ability. This may be related to lactic release and low pH levels after PLA degradation. As mentioned above, PCL scaffolds may offer more benefits for bone tissue repair. Hence, we used the PCL scaffold for the further in vitro study.

To enhance the biological activity of 3D printed PCL scaffolds, PDA was used to graft TGF-β_1_. The induction of osteogenic differentiation mainly depends on the support of growth factors, such as TGF-β_1_, which is one of the generally studied growth factors for the enhancement of bone repair in vitro and in vivo. Nevertheless, its actual response remains uncertain. No benefit to bone repair was observed with TGF-β_1_ concentration ranging from 0.01 to 0.1 ng/mL [68,69]. When the concentration of TGF-β_1_ exceeded 10 ng/mL, it aggregated with the expression of sulfated proteoglycans and aggrecan. However, 100 ng/mL of TGF-β_1_ reduced this osteogenic phenotype [70]. Our study showed a similar result. The ALP activity after MG-63 coculture was more prominent when the level of TGF-β_1_ was between 10 and 100 pg/mL (Figure 8). When the 3D printed scaffold interacted with 20 ng/mL TGF-β_1_, the average releasing level of TGF-β_1_ was almost 100 pg/mL (Figure 7). Hence, we concluded that the best TGF-β_1_ concentration for interaction with 3D printed PCL scaffold was 20 ng/mL compared with 10 ng/mL. The results showed that PDA could effectively immobilize TGF-β_1_ on scaffolds. Moreover, they demonstrated improved cell transformation and adhesion after TGF-β_1_ and PDA modification. 

A biocompatible material is defined as a material that does not release any toxic matter and supports cell growth that can be evaluated by an MTT assay [71]. The MTT assay in our study showed that PDA and TGF-β_1_ products were not only nontoxic agents, but also promoted cell growth. ALP activity, an initial indicator for functionality and differentiation of osteoblast, was measured by the ALP activity assay. The ALP enzyme released by osteo-differentiating cells is important for bone regeneration because it enhances inorganic phosphate generation, which is important for bone formation and mineralization [72,73]. The basic ALP level in the PCL scaffold was higher than that in another reported traditional manufacturing PCL scaffold [74]. This may indicate a better designed framework can offer a better environment for cell growth with the 3D printing technique. The greater presence of ALP activity was noted in cells cultured with the PCL-TGF-β_1_ scaffold compared with the pristine PCL and PCL-PDA groups. The ALP study also proved that PDA and TGF-β_1_ coating did not affect the activity of osteoblasts. This result also suggests that the PCL scaffolds with TGF-β_1_ grafting supported the osteo-differentiation of cultured cells better. 

## 5. Conclusions

We designed the 3D printed TGF-β_1_-immobilized PDA-grafted PCL scaffold for bone tissue regeneration. Well-interconnected microporous constructions in terms of physical properties were present in the 3D-printed PLA and PCL scaffolds, and they also exhibited satisfactory cell adhesion in vitro. In addition, the PDA coating affected the surface properties of the 3D printed PCL scaffolds and promoted the sustained release and grafting of TGF-β_1_. The constant release of TGF-β_1_ was helpful for osteogenesis differentiation and cell proliferation. In consequence, the PDA coating method can be applied as an ideal procedure to fix TGF-β_1_ onto a 3D-printed scaffold, allowing bone repair in the 3D printed scaffolds with exceptional biocompatibility, enhance cellular growth, and has potential as a stem cell delivery system the biological engineering. However, further in vivo studies are necessary for greater understanding of the mechanism of bone regeneration.

## Figures and Tables

**Table 1:** Pore size and line width of 3D printed PLA and PCL scaffold (*n* = 5).

	Pore Size (μm)	Line Width (μm)
3D printed PLA	684 ± 66	246 ± 34
3D printed PCL	682 ± 50	277 ± 36
